# Extracting spatial knowledge from track and field broadcasts for monocular 3D human pose estimation

**DOI:** 10.1038/s41598-023-41142-0

**Published:** 2023-08-28

**Authors:** Tobias Baumgartner, Benjamin Paassen, Stefanie Klatt

**Affiliations:** 1 Institute for Exercise Training and Sport Informatics, German Sport University, Cologne, Germany; 2 German Research Center for Artificial Intelligence, Berlin, Germany

**Keywords:** Information technology, Diagnostic markers, Computational science

## Abstract

Collecting large datasets for investigations into human locomotion is an expensive and labor-intensive process. Methods for 3D human pose estimation in the wild are becoming increasingly accurate and could soon be sufficient to assist with the collection of datasets for analysis into running kinematics from TV broadcast data. In the domain of biomechanical research, small differences in 3D angles play an important role. More precisely, the error margins of the data collection process need to be smaller than the expected variation between athletes. In this work, we propose a method to infer the global geometry of track and field stadium recordings using lane demarcations. By projecting estimated 3D skeletons back into the image using this global geometry, we show that current state-of-the-art 3D human pose estimation methods are not (yet) accurate enough to be used in kinematics research.

## Introduction

Applying human pose estimation (HPE) in sports has steadily been gaining popularity. A recent review shows a plethora of possible applications^1^. These range from estimating typical gait parameters^2^, over “bad pose” detection^3^ to martial arts training^4^. While there are a number of publications that demonstrate remarkable performance in recognizing specific actions, not much research has been conducted into applying these methods for data acquisition towards research in sports sciences. For instance, using large amounts of kinematic data from world-class runners, we could work towards answering questions like: What are the hallmarks of great running form? This seemingly fundamental question has not been completely answered yet^5^, as more research into the whole-body movement patterns of running is necessary.

There is a wide variance in human locomotion and there is no *single* correct running form or technique^5,6^. Nevertheless, running kinematics have been shown to account for up to 94% of the variance in running economy^7^ for novice athletes. So far, there is no unified model for determining running efficiency and evaluation of running kinematics. Instead, the literature deals with isolated aspects of the running form like heel velocity or thigh extension angle^8^. To make more generalized statements, and build a more holistic running model, we would need large high-detail datasets of running kinematics. The conventional kinematic lab setup is very costly and time-intensive^9^. It uses markers that are attached to the athlete and uses very high-rate, high-precision cameras. While there are approaches to validate in-lab vision systems^10^, the limitations of the lab setting remain: It is hard to acquire world-class athletes to undergo these investigations and virtually impossible to collect a large enough data set to account for all possible variability between athletes.

On the other hand, a purely vision-based approach for collecting the same data could be a cheap and highly scalable alternative. Such an approach, however, would need to be validated against gold-standard measurements. If reliable enough, it would allow leveraging existing recordings from TV broadcasts to collect large-scale datasets. Due to the dense history of recordings, it would be possible to perform long-term studies on individual athletes to monitor their changes in kinematics over time as well as over the course of a single race. Factors like fatigue in combination with pacing and race strategy will become possible to study, which are not accessible in a lab setting.

In order to validate and use 3D human pose estimation in the wild as a research tool for sports science, there is fortunately a large subset of running events that should allow the derivation of additional information from existing footage: races in a track and field stadium. Lane demarcations, as well as additional starting block and finish line markers, are standardized between venues. Using these markers, it is possible to triangulate the camera in the stadium and thus reconstruct the full 3D scene.

In this paper, we demonstrate that it is possible to extract 3D scene information from ordinary (and historical) track and field recordings. We programmatically construct all possible extrinsic and intrinsic camera parameters for a given frame. Furthermore, we find the exact scene geometry for a range of test video sequences. Using this actual scene geometry, we demonstrate shortcomings and opportunities of current monocular 3D HPE methods.

**Figure 1:**
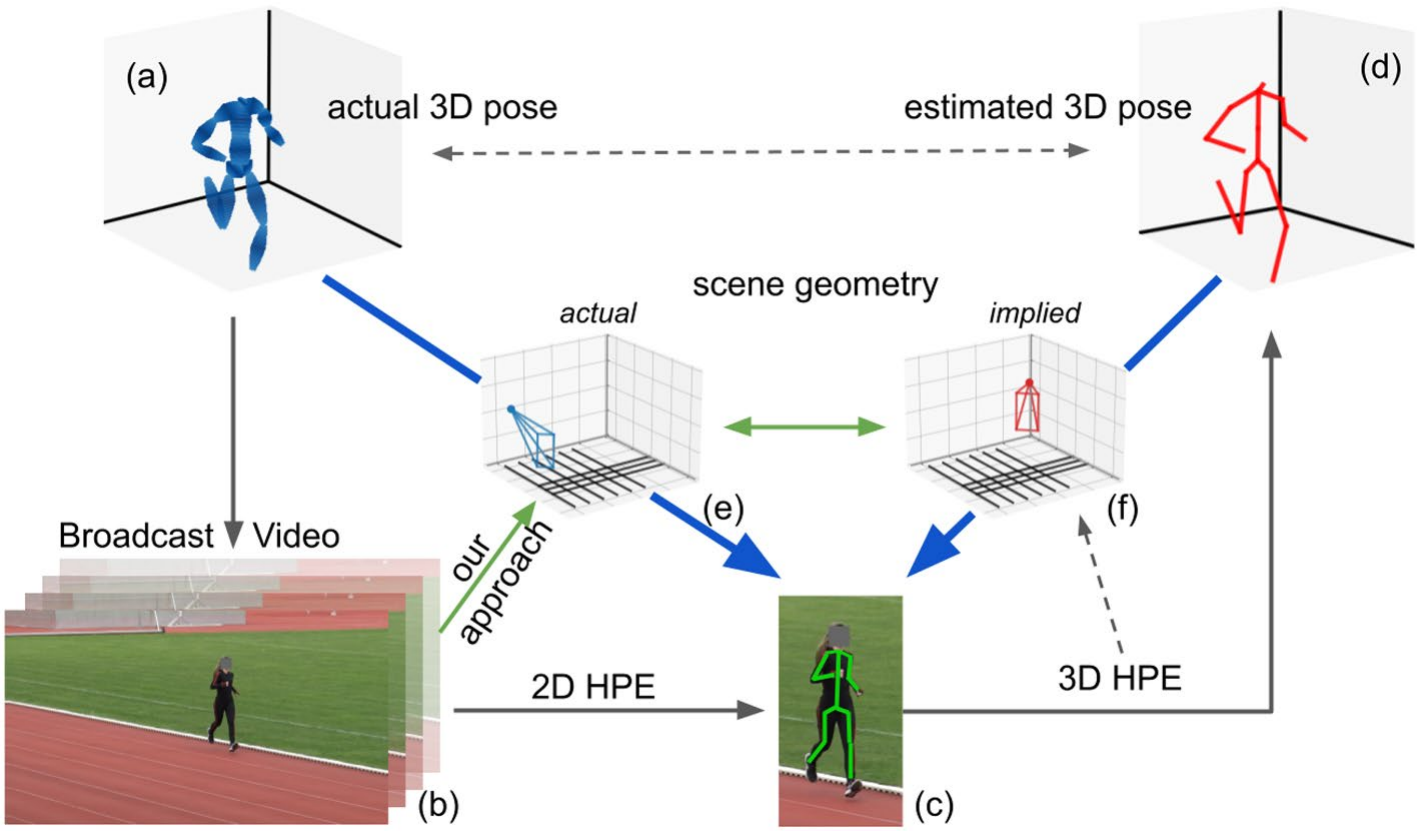
Overview of common 3D human pose estimation (HPE) process. The actual real-world 3D skeleton of the athlete (**a**) is projected into image (**b**). During this step, the actual scene geometry (camera orientation and location w.r.t. athlete) (**e**) is lost. A 2D skeleton can be reliably annotated or estimated via 2D HPE (c). Recovering a 3D pose (**d**) from the 2D joint locations implies a certain geometry (**f**). Both combinations of actual pose plus geometry (**a**) &(**e**) and estimated pose plus implied geometry (**d**) &(**f**) result in the same 2D joint location projection (**c**) (*cf*. thick blue arrows).

Fig. 1 illustrates the common 3D human pose estimation process. During capture, the actual skeleton (a) is projected into the TV broadcast footage (b), removing 3D information about the world (e). Using a 2D skeleton (c) to estimate a 3D pose (d) implies a certain 3D geometry (f) or even leverages explicit 3D assumptions about the scene. Skeletons from 2D HPE are both consistent with the actual 3D pose and geometry (a)$\circ$(e)=(c), as well as the estimated pose and implied geometry (d)$\circ$(f)=(c) ($\circ$-operator: “project skeleton using geometry”). If the implied geometry was correct (and thereby equal to the actual geometry), then the estimated pose would exactly match the actual 3D pose. In our experiments, we measure the deviation between (d)$\circ$(e) and (c). If the actual 3D pose (a) is equal to the estimated 3D pose (d), then (d)$\circ$(e)=(c). However, we find that (d)$\circ$(e) and (c) deviate significantly. We deduce that the expected error between the *estimated* 3D pose and the *actual* 3D pose make its usage infeasible for kinematic research.

The main contributions of this work are: (1) We demonstrate that there is a significant error when projecting monocular 3D HPE into 2D for current methods in a scenario that has not been observed in training Section "Experiments". (2) For this, we develop a novel method to uncover *all* candidate scene geometries based only on knowledge about a *single* vanishing point - Section "Method". (3) We arrive at a single degree of freedom to pick 3D geometry and manually annotate 158 frames of international elite athlete performances. (4) We show that current monocular 3D HPE methods (even when trained on additional data) do not generalize towards this data.

## Related work

*Monocular 3D human pose estimation (HPE)* methods act on flattened information about the world: In the process of taking the picture, the actual 3D world coordinates are projected onto the image plane in 2D. Recovering a 3D human pose from this 2D information implies that this lost information is attempted to be recovered from the scene (*cf*.  Fig. 1). For 2D HPE, it is possible to manually annotate existing imagery without access to the original recording process. The most common datasets and benchmarks use arbitrary publicly available images and densely label them using crowd-sourcing^11,12^. Using these datasets and competing on these benchmarks, the current state-of-the-art methods in 2D HPE perform ever closer to human-level performance^13–15^. In contrast, for 3D HPE, ground-truth pose data needs to be recorded for the training data, requiring a more complex lab setup. For example, the *human3.6m* dataset consists of a set of 11 actors in 17 different scenarios, recorded with 4 synchronized cameras and marker-based motion-capture^16^.

One approach for 3D HPE is based on video data. If a system can track a 2D skeleton over several subsequent frames, it can combine these frames and solve the combined task of predicting a 3D skeleton that explains all the 2D projections of the humans in the scene^17–19^. There is a vast body of works in this vein, solving for consistency leveraging limb length^17^, realistic movement^20^, or temporal context^18^. Another class of methods attempts to solve the problem of recovering the 3D information of the human poses by simultaneously predicting the depth of the current image^21–23^. These methods do not necessarily need to be combined with video information. By predicting the distance of each object, person, and surface in the frame to the camera, these methods construct a so-called 2.5D image that can be combined with 2D HPE to derive 3D poses. Modern approaches solve these two tasks simultaneously, inducing a bias on the possible configurations of the 3D skeleton. In their study^21^, Sarandi et al. additionally model the intrinsic camera parameters to arrive at a state-of-the-art system for monocular 3D HPE in the wild (in the following referred to as *MeTRAbs*).

To estimate camera parameters, one can also use sports-specific domain knowledge^24–27^. These methods match a known court or field template against the visible image to derive a projection transform. In particular, these templates contain corners and orthogonal lines, which makes distinct vanishing points identifiable. By contrast, our method derives the 3D-geometry of the scene using only lose constraints of parallel lines (with known distance), using a single vanishing point and temporal consistency.

*Computer vision for kinematics in sports* Within the scope of current research on gait and running kinematics, machine learning methods find more and more applications for data collection^28^ and uncovering signals in large datasets^29,30^. There is a large variation in running execution between subjects^5,6^. Thus, studying the whole-body kinematics of running and uncovering signal beyond the comparison of single aspects requires combined datasets of many runners. It is well-known that recognizing particular features in gait requires large amounts of data^31,32^.

Xu et al.^31^ experimented with a combination of kinematic (movement) and kinetic (forces) data to try and understand differences in the gait patterns of low- and high-mileage runners. They find that the ankle and knee carry signal in the sagittal plane (orthogonal view to movement direction) of the runner. Using a dataset on the order of 100s of runners, they train a neural net to classify into different discrete categories of weekly mileage and then analyze the learned weights. However, the authors acknowledge that the high variation in gait patterns makes it infeasible to infer any higher-order statements beyond predicting the weekly mileage.

There are problems in translating lab findings regarding running on a treadmill to the wild. For example, independent investigations measuring the knee angle progression between indoor and outdoor running showed differences of 2–3$^{\circ }$^33,34^. Even such minor angle differences can change running economy for novice athletes^7^. Rendos et al.^9^ show that in triathletes, there is a significant difference of  2–4$^{\circ }$ in knee flexion between warm-up and during the transition phase of a race. Overall, these findings indicate that running is best studied in the field and that high angular accuracy (below 3$^\circ$ error) is required. This motivates our study: We aim to validate the accuracy of monocular 3D HPE from TV recordings of running in the field.

*Camera calibration and sport-field registration* Calibrating the camera from existing broadcast footage has been used to analyze sporting events. The following works use the fact that the dimensions and line markings are well-known for ball sports: Chen and Little generate synthetic data with known 2D–3D correspondences to train a Siamese network that roughly predicts the orientation of a fixed camera. They refine the initial guess using the differences between the simulated scene and edges in the actual image^35^. Chu et al.^36^ expand upon this idea by allowing for sparse keypoints instead of complete line detections. Theiner and Ewerth propose a method that solves the task in a single shot instead of refining an initial guess^37^. All of these methods first detect keypoints and corners of known lines in the image and then calculate a homography that maps the image points to a 3D scene, which in turn determines the absolute position of the camera in the stadium. In our scenario, such approaches are not applicable, because we only know the distance between parallel lines, but do not exactly at which position in the stadium the camera points. Additionally, we only use lines that are all parallel to each other. Because of the resulting co-linearity of our input data, a homography cannot be computed. Instead, we solve two subtasks, namely *lane detection* and *vanishing points*.

*Lane detection* The classic line segment detection algorithm is the *Hough Line Transforms*^38^, which uses a voting scheme over all image pixels to determine lines for some specific threshold. This method is prone to errors for changing image conditions and is computationally expensive. Modern approaches are still computationally complex, but are geared towards parallel execution on GPUs: Dai et al.^39^ use a convolutional neural network to solve the task. Xu et al.^40^ use the self-attention mechanisms of the recent transformer architecture to improve upon the challenge of selecting the right thresholds. Li et al.^41^ expand prior methods to also work reliably for images with large lens distortions. Line segments can now be combined in a semantically meaningful way to infer the running lane markers. Our approach closely reflects the work of Mammeri et al.^42^, who propose a method based on the Hough transform and tracking over time for consistency.

*Vanishing points* Lastly, in our approach, we combine the detected lanes to then infer the main vanishing point. This vanishing point, in combination with the lanes, is the sole basis for our calibration approach. Like with the other steps above, there are some popular deep learning approaches. We experimented with the state-of-the-art approach by Zhou et al.^43^. Instead of the typical Hough voting scheme, they perform a transform into a conic space in which they directly aggregate information about vanishing points, while virtually skipping the line segment portion of the method. While this method performs exceptionally well on its target benchmarks, we observed some minor problems and deviations in our data: Lines pointing towards the resulting vanishing points did not align with the visible lane markers. We therefore stuck to an approach similar to Mammeri et al.^42^.

## Method

**Figure 2:**
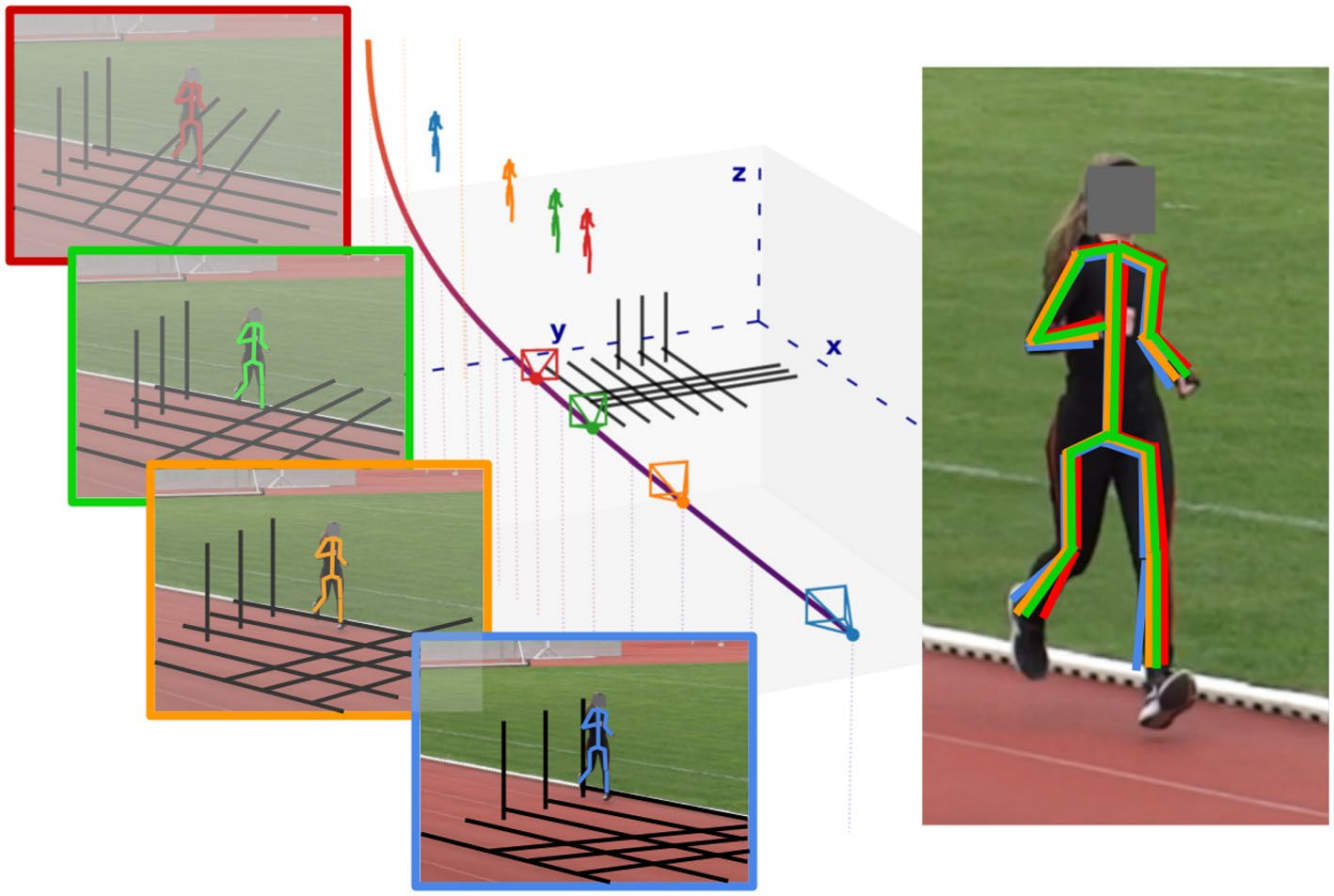
Multiple plausible camera parameters for a single frame. The color-graded curve shows all possible parameters, and the resulting relative camera location, consistent with the lane vanishing point. Four of these parameter sets and their 2D/3D rendering are shown (color-matched). Left: 2D Projection of scene with lanes (black) and skeleton. Center: 3D scene with 3D skeleton located consistent with the 2D Projection. Right: Overlay of all 2D projected skeletons for the respective geometries. Correct skeleton from 2D HPE in dashed black. *Best viewed in color*.

In the following, we first introduce our line of argument and then give details on the extraction method that we use to show the shortcomings of current methods and opportunities for future approaches. We introduce a method that extracts a dense set of possible camera configurations that are consistent with the lanes in a given scene from just a single vanishing point (*cf*.  Fig. 2).

Our method works solely from the knowledge of a single vanishing point. From there, we determine the extrinsic camera parameters for rotation ${\textbf{R}} = {\textbf{R}}_x \cdot {\textbf{R}}_y \cdot {\textbf{R}}_z \in {\mathbb {R}}^{3\times 3}$ (camera azimuth, elevation and roll), as well as the location of the camera with respect to the scene ${\textbf{t}}\in {\mathbb {R}}^{3\times 1}$. We assume to have square pixels, no skew and define the principal point of our virtual camera in the exact center of the frame, thereby leaving only the field-of-view as free intrinsic parameter. We determine the field-of-view and create the intrinsic camera matrix ${\textbf{K}} \in {\mathbb {R}}^{3\times 3}$ and overall projection matrix ${\textbf{P}} = {\textbf{K}} \cdot [{\textbf{R}} | {\textbf{t}}] \in {\mathbb {R}}^{3\times 4}$. To project a 3D point (*X*, *Y*, *Z*) back into pixel coordinates (*x*, *y*), we compute:

1 $$\begin{aligned} (x, y) = (\frac{u}{w}, \frac{v}{w}), \quad \text { where } (u, v, w) = (X, Y, Z, 1) \cdot {\textbf{P}} \end{aligned}$$

The last remaining factor about the broadcast camera that this model has not taken into account is the potential lens distortion. We show that this distortion is negligible to our argument in the supplementary material.

**Figure 3:**
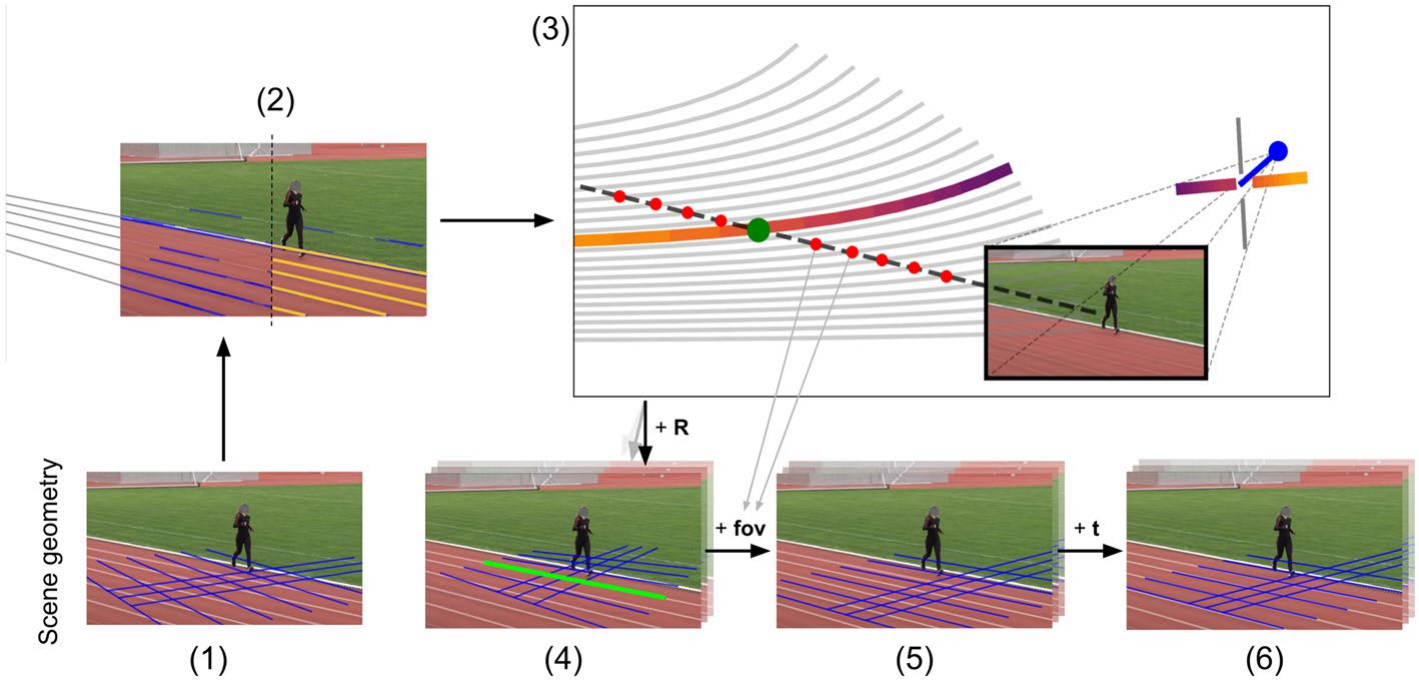
Overview of our approach. In the bottom row (**1**, **4**, **5**, **6**), the current best guess for the scene geometry is indicated in blue. In (**2**) and (**3**) we illustrate the computation and processing steps. See text for details.

Fig. 3 illustrates our approach as a flow-chart. The bottom row shows the current estimate of the scene geometry after the respective steps. Each of the steps in the following listing refers to the subfigures with the same letter Fig. 3(1)–(6).

1. We start with a random guess of the scene geometry. As can be seen, the white lane demarcations and the blue projection of the estimated scene geometry do not align at all (*cf*. Fig. 3(1)).
2. We first extract straight-line segments from the image using a linear Hough transform (left half, blue segments) and cluster these line segments into the main track lanes ${L_j}$ (right half, yellow lines). We determine the main vanishing point $v_0$ as the intersection of the lanes $L_j$ (indicated by gray lines Fig. 3(2)).
3. Next, we determine a dense set of possible azimuth and elevation pairs that are consistent with the vanishing point $v_0$. Fig. 3(3) shows elevation-isolines. Each of the curves (gray and gradient) has a fixed elevation and changes in the camera azimuth lead to the indicated movement of the simulated vanishing point. As the blue camera in Fig. 3(3) pans from left to right (purple to yellow), the vanishing point shifts from right to left and moves along the indicated line. Each intersection (red dots) on the line between the image center and $v_0$ (dashed line) describes a candidate pair of elevation and azimuth. Each of the azimuth/elevation pairs defines a camera *C* (*cf*. Fig. 3(3)).
4. We compute the roll of the camera using the assumption that the horizon line is horizontal. We thereby determined the rotation matrix ${\textbf{R}}^C$ and update the scene geometry estimation for each of the candidate cameras *C*. Observe, that in Fig. 3(4), the scene geometry lines partly point in the correct direction (central green line). The surrounding blue lines are not consistent with the visible image yet, due to an incorrect field of view (fov). The fov and intrinsic matrix ${\textbf{K}}^C$ can be determined using the distance of the red intersection points in Fig. 3(3) from the target vanishing point $v_0$. We adapt ${\textbf{K}}^C$ to shift the simulated vanishing point onto $v_0$ (*cf*. Fig. 3(4)).
5. This results in a scene geometry that matches $v_0$, which means that projected parallel lines are in line with the lane demarcations on the track. There is still some offset between the calculated scene geometry and determined lanes though. In order to manipulate the width of the projected lines, we adapt the distance *d* of the camera and shift the camera itself to perfectly line up with the image. Using *d*, the view target of the camera, and the rotation ${\textbf{R}}^C$, we can ray-trace the exact location of our simulated camera, and thereby the translation ${\textbf{t}}^C$ (*cf*. Fig. 3(5)).
6. Finally, we compute the overall projection matrix ${\textbf{P}}^C = {\textbf{K}}^C \cdot [{\textbf{R}}^C | {\textbf{t}}^C]$ and arrive at the candidate calibration depicted in Fig. 3(6).

As indicated in Fig. 1, for the estimated 3D pose to closely match the actual 3D pose, the implied geometry has to match the actual scene geometry. Readers can easily convince themselves of this by mentally rotating a 3D skeleton in front of a camera (which is equivalent to the camera moving around the object). Any change in camera azimuth, elevation, or roll will change the 2D projection of the skeleton. Therefore, in the reverse direction - lifting a 2D skeleton into a 3D scene - the 3D skeleton must also change with a moving camera in order to still be exactly projected onto the same fixed 2D skeleton. It follows, that a perfect 3D HPE method will implicitly use the correct scene geometry. We do not have access to the implied geometry of different monocular 3D HPE methods. But we can test single geometries, by projecting the estimated pose (*cf*.  Fig. 1d) using the actual geometry (e) into the image and comparing it to the original 2D skeleton (c). If this projection does not match the 2D skeleton, then the monocular 3D HPE method implied an incorrect scene geometry. Our experiments (*cf*. Section "Experiments") will investigate this aspect in more detail. As an auxiliary method, we also introduce a scheme to derive the actual geometry using the lane demarcations of the home stretch in a track and field stadium.

The lane demarcations point towards and intersect in vanishing point $v_0$, which is located somewhere outside the upper left corner of the image (*cf*.  Fig. 3(2)). Our method finds all sets of camera parameters that result in vanishing points ($v_0$, $v_1^k$), such that $v_0$ always matches the lanes and $v_1^k$ is the second vanishing point defined by lines orthogonal to the lane demarcations in 3D ($v_1^k$ is located somewhere to the upper right of the image). Fig. 2(left) shows 4 different exemplar geometries for the same $v_0$ and different $v_1^k$.

In Fig. 2, the effect on the 2D projection for different 3D geometries is shown. It demonstrates the difference between the same 3D skeleton rendered using different camera locations, which are all consistent with the visible lanes. For this, we construct a minimal 3D scene (*cf*.  Fig. 2, left, black grid lines) with typical track and field dimensions. Simulating a pinhole camera and the rendering process, we can limit the possible set of camera parameters to a line in 3D space (*cf*.  Fig. 2, color-graded curved line). Each of these camera configurations has a unique camera position that lies along the shown path. Rendering the black grid from the 3D scene (center) into a simulated camera and overlaying it on the original TV image results in the 4 different projections on the left (correct parameters=orange frame). For each of the 4 simulated cameras, we display the estimated absolute 3D positions of the athlete (middle). Using an off-the-shelf monocular 3D HPE method, we place the athlete in the scene and project the predicted 3D skeletons back into 2D. The same 3D pose results in the 4 different 2D projections (right), depending on the implied geometry of the scene. The original 2D HPE is shown in black/dashed. Observe that the reprojection of the correct geometry (orange) differs from the correct 2D skeleton as well.

### Extract geometry

Our proposed method allows for extracting the scene geometry and camera parameters from an image with typical track and field lane markings. For our approach, we make the following assumptions: pinhole camera; no digital zoom; fixed camera location (*i.e*. , no spider cam); the two main vanishing points are horizontal; 2D human pose estimation works perfectly. These assumptions are empirically validated in the supplementary material.

Our method falls somewhere between an analytical and constructive grid search approach. We discuss in Section "Discussion" why this was preferable to a gradient-based method in this use case. Below, we first describe the impact on $v_0$ of changing different parameters in the simulation pipeline and then work backward from these changes to step-wise construct dense parameter sets that all explain the current image. For this, we first make some general observations about the rendering process below. The parameters of our rendering pipeline are: camera position (x, y, z), camera orientation (azimuth, elevation, roll), field-of-view *fov*, and focal length *F*.

Due to the heavy markings on the ground in track and field stadiums, we can easily determine a vanishing point $v_0$ from the image using only low-level computer vision methods, namely, a Hough transform. In order to match $v_0$, we reverse the rendering process, by investigating each free parameter of the camera and reporting its impact on the displacement of $v_0$.

We define the *main* vanishing point of the scene as the point where the running lanes intersect (*cf*. Fig. 3 (2, 3)). To find this point, we extract line-segments from the image using a *Hough Line Transform* (default OpenCV implementation, https://opencv.org/). We cluster the resulting line segments and filter down to only segments that roughly point in the same direction. We further combine line segments by their exact image angles to arrive at an approximation for the running lanes. Combining the pairwise intersections of these lanes and performing a majority vote leads to the frame vanishing point $v_0$. In the supplementary material, we investigate the impact that lens distortion could have on our method.

*Camera position* (${\textbf{t}}$). Basic optics tells us that moving the camera without changing its direction results in the same vanishing point.

*Camera orientation* (${\textbf{R}}$). In Fig. 3(3) we show how the vanishing point moves, when panning (azimuth) and tilting (elevation) the camera: Fig. 3(3) Let the green dot be the vanishing point of the lane demarcations. Keeping all other camera parameters fixed, if we pan the camera (blue dot) to the left, the vanishing point will move to the right and curve up. The thick graded line shows the course of the vanishing point as the camera’s azimuth changes from 89$^{\circ }$ (darkest) to 1$^{\circ }$ (brightest). Gray curves show the progression of changing azimuth for varying values of elevation.

*Field-of-view and focal length* (${\textbf{K}}$). Both field-of-view and focal length change the dimensions of the image plane relative to the scene. Keeping the camera position and orientation fixed, a change in these parameters thus means a larger or smaller crop of an infinitely sized image plane surface. Therefore, the vanishing points move in a straight line away and towards the center of the image (principal point) when varying either *fov* or *F*. Without loss of generality, we keep *F* fixed going forward and only consider changes in *fov*. The dashed black line in Fig. 3(3) indicates the shift of the vanishing point when changing *fov*. Each intersection of the dashed black line with an elevation-isoline defines a pair of azimuth/elevation that can explain the present vanishing point.

For the following experiments, we manually pick the correct geometry out of the resulting set of possible options using all of the above additional visual clues.

## Experiments

In this section, we use our method for determining the scene geometry (*cf*. Section "Method") to demonstrate that current state-of-the-art monocular 3D human pose estimation methods do not accurately account for the underlying scene geometry, resulting in significant discrepancies between the estimated 3D poses and the actual 3D poses. We demonstrate this discrepancy by swapping the implied scene geometry for the approximately correct geometry (using our method) and recording the resulting differences in the projected image (*cf*. Fig. 1).

In this experiment, we determine one fixed 2D skeleton for each of the evaluated frames/athletes. This 2D HPE method is based on a ResNet-50 backbone, trained on MPII^12,21^. We lift each 2D pose into 3D using different monocular 3D HPE methods (see below). We place the resulting 3D skeletons into a simulated scene, and project this scene back into the 2D image, using the actual scene geometry, as determined with our method. We can now compare this reprojection to the original 2D skeleton.

In terms of Fig. 1: We take a 2D skeleton (c) and lift it into a 3D skeleton (d). This monocular 3D HPE process implied some unknown geometry (f). We use our method to determine the actual geometry of scene (e) and project the 3D skeleton (d) back into 2D image (c) using this correct geometry. We show that the resulting reprojection differs from the original 2D skeleton, which in turn must mean that the implied geometry (f) differs from the actual geometry (e) and that the estimated 3D pose (d) differs from the actual 3D pose (a).

We quantify the reprojection error and estimate the underlying 3D error that caused the reprojection error. We additionally provide evidence from a small real-world experiment that both our method closely approximates the correct scene geometry and that our approximation of implied 3D knee angle errors is reasonable.

We also provide additional adversarial experiments in the supplementary material that demonstrate that the below results are not just artifacts of the limited pinhole camera model. Lastly, we also copy the typical scene setup at our local track and compare our method to laser-verified groundtruth measurements in Section "Ground truth evaluation".

### Annotations

We annotate frames of five video sequences from different venues, athletes, and distances from major broadcast athletic events (*e.g*. Olympic Games, Diamond League, ...). For these, we manually go through all the frames to ensure that our calculated results are consistent with all visible clues in the scene. Using lane demarcations, our algorithm automatically generates an exhaustive set of candidate camera parameters. We can then determine the correct camera parameters for every single frame using an annotation tool that lets the annotator slide through the various plausible camera parameters until they perfectly align with all additional visual clues. Using the resulting scene geometry of each frame, we ray-trace the exact 3D location of the athlete whenever they touch the ground. We determine the frames that depict the touch-down phase of the athletes’ stride by analyzing the foot progression of the 2D human pose estimate. We scale the 3D skeleton and additionally scale and translate the 2D projection to minimize the distance to the original 2D pose. We only use athletes that are fully visible to avoid errors due to occlusions from other athletes. This process results in a total of 355 frames, which we evaluate in the following.

### Evaluated monocular 3D HPE methods

We compare 3 state-of-the-art methods for monocular 3D HPE: Strided Transformers^18^, RIE^17^ and MeTRAbs^21^. While the former two methods are solely trained on Human3.6m^16^, MeTRAbs is additionally trained on external data and purposely build for 3D HPE in the wild. For all these algorithms, we run 3D HPE, then detect the absolute position of an athlete’s foot in the scene, and place the 3D skeleton at that location. The predicted orientation and scale of the 3D skeletons depend on the 2D/3D correspondences in the training data. As the 3D skeletons do not necessarily comply with the actual geometry of the scene and the orientation of the camera, we adjust the scale to match the height of the projection. We also align the orientation of the predicted 3D skeleton with the axes of the constructed scene (*cf*.  Fig. 2).

A preliminary analysis showed that MeTRAbs has superior performance over the other two methods. We furthermore compare MeTRAbs to slightly improved versions of itself. We inject information into the base algorithm that is ordinarily not available to it. The purpose of these modifications is to show that there is still an offset between the projection of the 3D skeleton and the actual 2D HPE in the original image, even when we improve the method by leveraging additional domain and scene knowledge.

*MeTRAbs + movement knowledge*. We exclusively investigate running footage in which the athletes run down the home stretch. We, therefore, know that the 3D skeletons in the scene should always face in the same direction and are moving in a straight line. The pan motion of the camera following the athlete impacts the relative orientation of the athlete to the camera. This often results in the 3D lifting portion of monocular 3D HPE to describe a curved trajectory. Straightening out the athlete’s path leads to a first improvement, leveraging domain knowledge about the scene.

*MeTRAbs + rotation knowledge*. Secondly, we compare the base algorithm to an improvement strategy in which we ideally rotate the 3D skeleton using the relative orientation of the camera to the skeleton. We use the same rationale as before: The athletes should always be facing the same direction. Only this time, we directly place and rotate the athlete such that they are facing the finishing line. We can perform this rotation of the skeleton because we know where the camera is located relative to the athlete using our method described and therefore again inject domain knowledge.

Both of these improvements leverage information that is not available to the base algorithm.

### Evaluation metrics

Ideally, for a perfect 3D HPE algorithm, placing the 3D skeleton in the correctly derived global geometry of the scene and then projecting it into the image using the derived camera parameters should result in a perfect overlap of the 2D skeleton and the reprojected 3D skeleton. Realistically, there will always be some margin of error. In the following, we measure this error for existing state-of-the-art 3D HPE methods. We further investigate the expected error over a sample size of 16 athletes and videos from different camera angles and pan-zooming recordings resulting in 355 data points.

We do not have ground truth 3D HPE data for the investigated videos, so we cannot perform the typical analysis of 3D MPJPE (Mean Per Joint Position Error). Instead, the reprojection error as described above is expressed in the 2D image space. Additionally, for each of the studied athletes, we simulate a movement in their knee in 3D space and record the resulting changes in the projected image. Below is a detailed description of our evaluation metrics; the results can be found in Table 1. We consider 17 major joint locations, commonly used across HPE benchmarks: head, neck, chest, navel, pelvis, 2$\times$ shoulder, 2$\times$elbow, 2$\times$wrist, 2$\times$hip, 2$\times$knee and 2$\times$ankle.

*Reprojection error*. Using the uncovered 3D geometry of the scene, we project the 3D skeleton into the image and compute the average per-joint offset to the corresponding 2D skeleton in pixels. For this, we use the 17 default human3.6m joint definitions^16^. Additionally, since the exact scene geometry is known (track lanes are $1.22\pm 0.01$ m wide), we scale this value by the real-word versus pixel height of the athlete. This is not the correct joint distance in mm, but just an approximation incorporating the image scale. For a true distance measure, we would require ground truth 3D skeleton information. We include this measure as it more accurately accounts for the distance of the athlete to the camera and the camera’s zoom.

*2D knee error*. For kinematic investigations, we are not particularly interested in the absolute position of each of the joints, but rather their relation to each other. As motivated in Section "Related work", we want to investigate the knee angles of the athlete. Since we do not know the correct 3D skeletons or detailed running kinematics in our test data, we measure the knee angle error for the 2D poses.

*Approx. 3D knee error*. If we had an orthogonal view of the athlete’s knee, the visible 2D knee angle (and its error) would roughly correspond to the 3D knee angle. For increasingly steep angles of the camera towards the sagittal plane of the athlete though, this correspondence breaks. For larger values of the camera’s azimuth, 2D knee errors result in more severe actual knee errors. We approximate the 3D knee error by simulating movement in the predicted 3D skeleton’s knee and recording its effect on the 2D knee angle error. The measured 2D knee error is then scaled accordingly for each of the evaluated frames.

**Table 1:** Comparison of the expected errors for different state-of-the-art monocular 3D HPE methods. See text for details. Error analysis for 355 frames for 16 athletes over 5 different venues and distances. *Mean (Standard Deviation)*.

Method	Reprojection error	Reprojection error	2D knee error	Approximate 3D knee error
	[pixel]	[mm]	[degree]	[degree]
Strided Transformer^18^	10.97 (4.26)	82.33 (16.26)	40.31 (14.75)	49.44 (27.69)
RIE^17^	12.03 (4.84)	84.26 (20.74)	40.31 (20.19)	44.24 (26.92)
MeTRAbs^21^	4.75 (3.19)	42.97 (49.04)	10.31 (10.66)	13.38 (31.15)
+ movement knowledge	3.76 (2.85)	35.01 (46.62)	6.81 (8.55)	9.94 (28.54)
+ rotation knowledge	5.30 (4.20)	42.55 (49.05)	7.47 (7.41)	8.45 (13.19)

The best approximated 3D knee error in our comparison is 8.45$^{\circ }$ with a standard deviation of 13.19$^{\circ }$ (*cf*.  Table 1). This margin of error is larger than the levels of change for significant differences in running kinematics and implied running economy as detailed in the literature^5,7,9,31^ (*cf*.  Section "Related work"), rendering the current state-of-the-art methods infeasible for the collection of data towards kinematic investigations.

### Ground truth evaluation

We perform a small validation study using an Xsens motion capture suit (MVN Link, Xsens Technologies B.V., Enschede, Netherlands, https://www.xsens.com/products/mvn-analyze). This IMU-based motion capture system has been independently validated with angle errors of $< 2.6 \pm 1.5^{\circ }$^44^. We set up multiple camera locations in the stands close to the finish line, to match typical broadcast images and triangulate the cameras’ positions using both a laser range finder and optical methods. The image used in Figs. 1, 2 and 3 display a still from our own video recordings, which is representative of the positioning and settings of broadcast video. In our experiment, one athlete runs on the home stretch of the track and we simultaneously record 3D motion capture and video footage, performing the TV-typical camera operations: pan, tilt, and zoom (up to 30x). We use the method described in Section "Method" to extract possible camera parameters. For 50 frames, we manually pick the camera parameters that best align the projected 3D skeleton with the athlete’s image. Speaking in terms of Fig. 1, we recorded the actual 3D pose Fig. 1a, annotated scene geometry Fig. 1e, and filmed the skeleton Fig. 1c. We now compare these to the estimated pose Fig. 1d.

First, we evaluate how well our method predicts the actual geometry and location of the camera in the scene. Our model is based on a pinhole camera, whereas in reality, we filmed with a conventional camera with multiple lenses. We, therefore, cannot expect our method to find the exact position of the real camera, but only of a virtual camera. We find that the predicted camera location is within 5.5% of the correct camera position (w.r.t. the distance of the camera to the athlete). The average offset of the predicted camera to the actual camera in x/y/z direction is 1.75m/2.67m/0.72m (min: 0.09m/0.18m/0.01m, max: 4.48m/9.50m/2.05m). The athlete is at an average distance of 37.56m to the camera (min: 14.57m, max: 71.41m).

We next compare the 2D HPE for these 50 frames to the projection of the recorded 3D Xsens skeleton using the correct scene geometry, resulting in an RMSE of $7.56 \pm 3.75$ pixel, which equals $50.42 \pm 28.51$ mm.

Finally, we evaluate the 3D angle error in the knee and elbow between the recorded and estimated skeletons (*cf*.  Fig. 1a,d). The left/right knee have an average error of: $8.39 \pm 4.41^{\circ }$ / $7.94\pm 5.84^{\circ }$, which is in line with our approximated 3D error in Table 1. The left/right elbow has an average error of: $15.81\pm 7.80^\circ$ / $11.85 \pm 5.65^\circ$, yielding an overall expected error in 3D angle prediction of $11.00 \pm 5.93^\circ$.

## Discussion

In this investigation, we demonstrate a technique for discovering the extrinsic camera parameters for a contiguous video sequence in a track and field stadium. Using only low-level computer vision methods (Hough transform), we leverage the extensive markings on the ground to generate candidate camera parameters. Combining these for all frames under some consistency constraints allows for the reconstruction of the entire 3D scene progression in the video. In our experimental setting, we used the discovered camera parameters to project 3D human pose estimation predictions back into the image using the correct camera parameters and compare them to their predicted 2D HPE counterparts. The resulting expected margins of error in the knee angle for a small sample of track and field recordings are larger than the real-world variance to be expected in running kinematics. The errors surpass the level of significant differences between runners, which makes collecting data to study running kinematics impossible using only monocular video footage and the current state-of-the-art methods in 3D human pose estimation in the wild. We advocate for injecting the readily available information on 3D geometry into future iterations of monocular 3D HPE systems.

It is important to note, that our approach does not extend past the walls and lanes of a track and field stadium and is not generalizable. The objective here was not to solve the broad task of *roughly* estimating human poses in all kinds of situations, but instead, we aim towards *accurately* approximating human pose kinematics in a very narrow domain to produce data for downstream investigations. Using the vast amounts of data from freely available video documentation of world-class athletic racing events as well as historical data and long-term change progressions could open up the possibility of studying human locomotion, or at least allow for more accurate modeling. We would like readers to consider the untapped potential of computer vision as a research tool in sports science.

In our experiments in Section "Experiments", we improve the reprojection of an existing method by injecting some domain-specific knowledge into its result. We modify the monocular 3D HPE using the derived camera parameters. The evidence that there are still significant errors under this ideal utilization of the 3D information means that the monocular 3D HPE is not simply offset in translation, scale, and rotation from the correct 3D skeleton, but that it is inconsistent with the overall pose. A track and field-specific monocular 3D HPE method should, therefore, incorporate this 3D scene information, instead of just using it to correct its output.

A straightforward naïve implementation of this idea could be seen as an extension to the 2.5D HPE methods: Perform 2D HPE, find complete camera parameters, and ray-trace each of the 2D points into the scene. In 2.5D methods, a depth map is created for the image, describing the distance of each object and person to the camera. Using our method, we can recover the 3D geometry of the lanes, but not of any objects or people in the image. Since we can recover the overall scene geometry instead of the distance of people to the camera, this naïve approach might be considered a 2.75D method, albeit being limited to the track and field setting in scope.

On the surface, the task of determining the correct camera parameters using the vanishing point and some lines in the image looks like a prime example of a deep learning, gradient-based solution. In addition to the described method (*cf*.  Section "Method"), we successfully experimented with uncovering *some* set of extrinsic camera parameters employing differentiable rendering. The simulation pipeline described in Section "Method" can be completely implemented in TensorFlow (or any automatic differentiation engine of choice) and then optimized to match vanishing points and certain landmarks in the image, just like our approach. The shortcoming of such a method is that it will always find *some* solution, but not *all* the solutions. In our approach, we sample a dense but discrete number of possible parameter pairs. We sweep through the parameter space at a sampling rate of $0.5^{\circ }$ azimuth. Neighboring parameter sets can then be interpolated to arrive at a continuous representation for all possible camera parameters. With that, we created an interface that lets domain experts move a slider to adapt the camera height or location for the second vanishing point, while always adapting the camera parameters to display a projection of the 3D scene that is consistent with the current frame.

A clear limitation of our method is the projection model using a pinhole camera. In reality, cameras have multiple lenses that will distort the image and bend straight lines, which affects the basis of our method, the Hough transform. Additionally, the pinhole camera model assumes that we can draw straight lines from the scene through the image plane into the center of the camera, whereas in fact the lens will refract that camera beam and move the effective camera center closer to the focal point and scene. Our validation experiment in Section "Experiments" demonstrates that, while we can find the correct scene geometry, we ignore the more minute details of lens distortions, and thus our predicted 3D location is offset from the camera by an average of 5.5%. In the domain of interest, we assume that the distortion effect for the footage we are investigating is not too large, as the kind of shots we analyze use tele-zoom cameras.

We provide additional experiments in the supplementary material to further demonstrate this point. In the experiments, we adversarially optimize both the lens distortion and exact camera location (within the estimated 5.5% radius) to improve the results from Table 1. We run 200 separate optimizations in which the calculated parameters from our method are adapted to minimize the reprojection error. The adversarial experiments result in improvements in the reprojection error by 0.36±0.48 pixels (lens distortion) and 0.66±0.48 pixels (camera location) respectively. The limitation of our camera model are therefore not severe enough to explain the observed effect in reprojection error.

Even with this limitation, the statement of this paper persists. For a given vanishing point, we can find a set of camera parameters that allows rendering all scenes for varying second vanishing points. Picking the correct perspective out of these options by hand, we show that current monocular 3D HPE methods are not consistent with the scene geometry. Projecting the estimated 3D poses into the 2D image using the correct geometry yields large errors. Thus, the geometry that was implicitly used by the monocular 3D HPE differs from the actual geometry.

## Future work

This study is meant as a starting point for using computer vision methods to extract data from freely available videos that can be used in future investigations into running kinematics. Our method could already be used to start annotating data as it is. 2D human pose estimation is by its nature easy to annotate. All the necessary information to ideally solve this task is given in the image and answering the question: “Which pixel in the visible image is closest to the elbow joint” can be accurately solved using a few judgments from annotators. In order to perform the same task for 3D, additional information beyond a 2D pixel location is required. We designed our method in a way that allowed us to create an annotation tool that can be used to quickly adjust the correct scene geometry using a single slider. This same process can be repeated for a vast set of videos. Using the naïve approach mentioned in Section "Discussion", we could generate a large kinematic dataset. Of course, we would want to do better than that and reduce the manual annotation burden even further. Still, in order to be able to use data in a sports scientific investigation, a researcher will always have to manually be able to confirm and adjust some sample of the data to approximate the recording error.

An apparent shortcoming of our method is that it currently only works on the straight sections in the track and field stadium. A natural extension would be to consider all camera locations and viewpoints in the stadium since there are just as many ground markings in the curves, as well as other sporting events (*cf*. Section "Related work"). Such an extension should be developed using higher-level vision methods and, most likely, a deep learning approach similar to the current research line of neural radiance fields^45^. As mentioned throughout this paper, we advocate for developing monocular 3D HPE methods that incorporate the domain-specific knowledge we extract with our method. It is unlikely that this will result in a generalizable solution, but we can further improve domain-specific 3D human pose estimation to support investigations into large-scale running kinematics analysis.

### Supplementary Information

Supplementary Information.

## Data Availability

Our method builds on and analyses publicly available video data from professional middle-distance running events. A full list of utilized video material, as well as groundtruth data for the validation study in Section "Ground truth evaluation" is available from the corresponding author upon reasonable request.
