# Supplementary material for: Extracting spatial knowledge from track and field broadcasts for monocular 3D human pose estimation
Source: Sci Rep. 2023 Aug 28;13:14031. doi: 10.1038/s41598-023-41142-0 (PMC10462612; doi:10.1038/s41598-023-41142-0)
Supplement: Supplementary file 1 — Supplementary Information. [file 41598_2023_41142_MOESM1_ESM.pdf]

Extracting Spatial Knowledge from Track & Field Broadcasts for Monocular 3D Human Pose Estimation - Supplementary Material

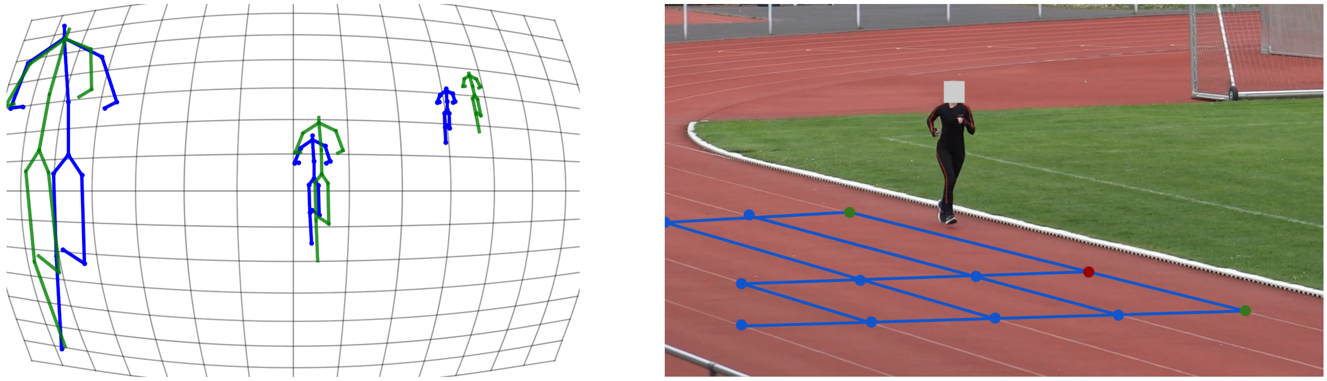

(a) (b)

Figure S1. (a) Effect of exaggerated lens distortion on the projection of the 3D human pose estimation. Grid-lines: Indication of distortion. Blue: Projection of 3D skeleton. Green: Distorted skeletons. (b) Calibration grid based on hurdle markers. Offset of marked points from a perfect grid: 0.65 ± 0.53 pixel. *Representative figure, not actually used data.*

1 Adversarial Experiments

In this section, we propose some further experiments to demonstrate that the errors in reprojection (*cf*. Tab. 1) were in fact not introduced by artifacts of our own method, but are baked into the methods (or rather the underlying training data). For this, we first investigate the impact that lens distortions could have on the reprojection. We furthermore test whether the imperfect camera calibration could account for entirety of the re-projection error.

1.1 Lens Distortion

In our pinhole camera model used so far, the only variable about the process of projecting the 3D world into pixels that we haven't touch on yet is lens distortion. In the above experiments, we used a simplified camera model that just ignores potential effects of lens distortion. Fig. S1a shows the potential error in re-projection for various locations and size, with an exaggerated lens distortion.

Manual Calibration To exemplify the low impact of lens distortion on our method, we perform a manual calibration of multiple frames of a single video (*cf*. Fig. S1b). For this we find frames that contain enough stadium markers. Fig. S1b contains markers for the locations of hurdles in the 100m, 110m and 400m hurdles disciplines. For this figure, we recreated the typical broadcast setup at our local track. Similar markings can be found in broadcast video and we perform the below calculations on actual broadcast footage. The locations of hurdle markings and therefore their relative positions are known. We perform the default camera calibration as provided by OpenCV (opencv-python==4.5.3.56, `cv2.calibrateCamera`) using these point correspondences. We extract the parameters for lens distortion from the resulting projection and apply the reverse distortion function to the image. Using this modified image, we perform the same steps of our method to determine the vanishing points as outlined in the Methods section. The resulting vanishing points differ from the original locations by less than 1%. The fact that lens distortion does not play an immensely important role in this scenario can also be seen in Fig. S1b: The line between the two green points perfectly overlaps with the white lane on the track. In fact, we measure the curvature of the lanes by measuring the distance of the red points to the line between the green points. We repeat this measurement for all pairs of points on the grid. The average deviation from the annotation pixel locations is 0.65 ± 0.53 pixels.

Lane Detection The first and fundamental step of our method is straight line detection. If the image contains a large amount of lens distortion, straight lines become curved and our method would not work (*cf*. Fig. S1a). As shown before, the lines in our scenario do not contain much curvature.

Re-projection Next, we want to investigate, whether lens distortion could have been the root cause for the re-projection errors displayed in Tab 1. Assume that the monocular 3D HPE methods in fact derived the correct 3D configuration and thereby correct implicit geometry (*cf*. Fig. 1f). If this was the case, then the re-projection error that we measured must stem from our imperfect camera model. To reject this notion, we setup an experiment in which we try to fit our 2D projections (using our 3D scene geometry) to the observed 2D image points solely using the formulation of lens distortion. We express the lens distortion as $x_{distorted} = x \cdot (1 + k_0 + k_1 \cdot r + k_2 \cdot r^2 + k_3 \cdot r^3)$. For comparison, Fig. S1a has parameters $k_0 = .4, k_1 = -0.15, k_2 = -0.35, k_3 = 0.15$. We now optimize the parameters $k_{0...3}$ for each 2D-3D skeleton pair individually, such that the re-projection error is minimized. In the process we allow for the re-projected skeleton to be freely shifted and scaled in size to best match the original 2D skeleton. Observe that in Fig. S1a the location and size of the re-projection can change due to lens distortion. We use the optimization framework in `scipy` using the *L-BFGS-B* optimization method[1]. For each frame, we run the experiment with 100 different initialization and chose the lowest resulting error. Overall, this improves the error of *Metrabs* by 0.36 ± 0.48 pixels or 3.18 ± 4.95 mm. We conclude that lens distortion does not solely account for the observed error in reprojection shown in Tab. 1.

1.2 Camera Calibration Error

As described in the real-world experiment in Sec. 4.3, our proposed method can be up to 5.5% off. In this next experiment, we investigate, whether this imprecision is the cause for the observed errors in Tab. 1. Like before, we start with the 3D scene geometry and projections extracted with our method. We use the resulting projection matrix $\mathbf{P}^C$ to project the 3D skeleton S^{3D} into 2D $S^{2D}_{proj} = \mathbf{P}^C \circ S^{3D}$ and compare it to the original 2D skeleton S^{2D}. We now setup an optimization problem to find some projection matrix $\mathbf{P}^\star$ that minimizes the reprojection error $|\mathbf{P}^\star \circ S^{3D} - S^{2D}|$. We restrict $\mathbf{P}^\star$ to describe a projection with a camera that is within a radius of 5.5% of the original distance to the scene. All other extrinsic and intrinsic parameters of projection $\mathbf{P}^\star$ are freely optimized. Like before, we use a *L-BFGS-B* optimization and take the optimum of 100 random initializations. The resulting projections are of course not necessarily consistent with the lanes in the scene anymore. On average, this relaxation improves the reprojection error by 0.66 ± 1.95 pixels or 5.79 ± 16.06 mm.

We know from our real-world experiments, that our predicted PC is less than 5.5% off the actual laser-measured scene geometry. We restrict $\mathbf{P}^\star$ in a way to be with 5.5% of PC. In Tab. 1 we show the expected re-projection error with current state-of-the-art methods (MeTRAbs: 4.75 pixels). If we improved the projection slightly (by less than 5.5%), we can improve upon this error by 0.66 pixels, which is, 14% of the expected error. In our experiment, we use this measure to demonstrate that current monocular 3D HPE methods have a larger error, at most 14% of which could be attributed to the probabilistic experimental setup. This still means that 86% of the error ($.86 \times 13.38^\circ = 11.5^\circ$) is due to incorrect implicit geometry. Even with the correction, this error would still make data collected in this way infeasible for further study in kinematic research. We argue that current methods need to be improved in order to allow for large-scale kinematic data collection and thereby eventually deeper insight into human locomotion.

2 The impact of imperfect 2D HPE

One assumption we made throughout this work is that 2D HPE works flawlessly and that its errors can be neglected. It is clear, that no probabilistic system ever performs perfectly. Let's assume though that we have an oracle 2D pose estimator (which by definition works perfectly). In our experiments, we compare methods that take 2D poses and lift these into 3D space. Whether or not the used 2D pose matches the exact pixel of the joint, does not change the fact, that the lifting process itself implies some geometry (*cf*. Fig. 1). Since this implied geometry is solely based on the collection of 2D skeletons, it is likely not exactly correct. We advocate for using clues in the image that allow us to derive the correct scene geometry and develop monocular 3D HPE lifts, that use this explicit geometry instead of implying it. In our experiments, we show that exchanging the implied with the correct geometry leads to reprojection error, which must in turn mean, that the implied geometry was not the same as the actual present geometry in a scene.

References

1. Zhu, C., Byrd, R. H., Lu, P. & Nocedal, J. Algorithm 778: L-bfgs-b: Fortran subroutines for large-scale bound-constrained optimization. *ACM Transactions on mathematical software (TOMS)* **23**, 550–560 (1997).
